# Molecular detection and characterization of *Anaplasma marginale* infecting cattle, buffalo, and camel populations in southern Egypt

**DOI:** 10.3389/fvets.2023.1169323

**Published:** 2023-05-11

**Authors:** Hassan Y. A. H. Mahmoud, Alsagher O. Ali, Tetsuya Tanaka

**Affiliations:** ^1^Division of Infectious Diseases, Department of Animal Medicine, Faculty of Veterinary Medicine, South Valley University, Qena, Egypt; ^2^Laboratory of Infectious Diseases, Joint Faculty of Veterinary Medicine, Kagoshima University, Kagoshima, Japan

**Keywords:** *Anaplasma marginal*e, cattle, buffalo, camel, Egypt, PCR, sequencing

## Abstract

Anaplasmosis is a severe tickborne disease of ruminants caused by *Anaplasma marginale*. *A. marginale* is distributed worldwide and attacks erythrocytes, resulting in an increased body temperature, anemia, jaundice, abortion, and, in some cases, death. Animals infected with this pathogen become lifelong carriers. In this study, we aimed to detect and characterize *A. marginale* isolated from cattle, buffalo, and camel populations using novel molecular techniques in southern Egypt. In total, 250 samples (from 100 cattle, 75 water buffaloes, and 75 camels) were analyzed by PCR for the presence of *Anaplasmataceae*, specifically *A. marginale*. The animals varied in breed, age, and gender, with most showing no signs of severe disease. By species, *A. marginale* was found in 61 out of 100 (61%) cattle, 9 out of 75 (12%) buffaloes, and only 5 out of 75 (6.66%) camels. All *A. marginale*-positive samples were examined for the heat-shock protein *groEL* gene and, additionally, for *major surface protein 4* (*msp4*) and *major surface protein 5* (*msp5*) genes to enhance specificity. Phylogenetic analysis of *A. marginale* targeted three genes (*groEL, msp4*, and *msp5*). This study provides the first report on using three genes for *A. marginale* detection in *Camelus dromedarius* in southern Egypt and generated new phylogenetic data for *A. marginale* infections in camels. *A. marginale infection* is endemic in different animal species in southern Egypt. Screening herds for *A. marginale* is recommended even when the signs of anaplasmosis are absent.

## 1. Introduction

Tickborne diseases are a serious challenge to global health. In Egypt alone, they pose a significant threat to animal health, in particular to local exotic and crossbred cattle and buffalo, thus potentially undermining the livelihoods of their owners (1). *Anaplasma* species are the most common tickborne pathogens in cattle and are endemic across six continents with a high incidence in tropical and subtropical areas of the world (2). The disease they cause is termed anaplasmosis, which is particularly common in ruminants (3). Among *Anaplasma* species (Rickettsiales: Anaplasmataceae), *Anaplasma marginale (A. marginale)* may be the most dangerous.

Anaplasmosis causes progressive hemolytic anemia and significant economic losses in tropical and subtropical areas (4). Ticks are known carriers of *A. marginale*, and approximately 20 tick species have been implicated as vectors of anaplasmosis (5). Bovine anaplasmosis is an economically devastating disease that results in losses to the dairy and beef industries through reduced milk production, weight loss, abortion, jaundice, and sometimes death (6, 7). The disease is mainly spread to cattle by ixodid ticks, but other routes of infection include fly bites and blood-stained objects, such as needles, ear tags, and castration equipment. Placental transmission may feature in the disease's epidemiology in specific areas (8). Fever, anemia, weakness, enlarged lymph nodes, abortion, reduced milk production, and jaundice are signs of anaplasmosis in cattle, and the disease can be fatal in severe cases (9). Cattle recovering from acute infection remain carriers for the rest of their lives and can act as sources of infection for previously naïve livestock populations, triggering endemic infection or epizootics (10).

The camel is a multipurpose animal playing crucial roles in the transport and provision of milk and meat in arid and semi-arid regions of the world. Although camels are hardy animals and can withstand the harsh conditions of dry areas due to their unique, adaptive physiology, their health can be adversely affected by a range of specific diseases (11, 12), including those transmitted by bloodborne parasites. Such diseases can cause anemia, emaciation, and even death in severe cases when camels are infected (13). Camel anaplasmosis has been reported as a subclinical disease in dromedary camels of Tunisia, India, and Saudi Arabia (14).

*Anaplasma* species are longevous microorganisms, potentially surviving in hosts for months or years, and the consequences of this phenomenon include increased transmission and the occurrence of new anaplasmosis outbreaks (15). Control measures include frequent surveillance, prompt treatment, and eradication of arthropod vectors, and their feasibility depends on several variables, including geographic location, husbandry practices, and implementation costs (encompassing items such as the vaccine or antibiotic treatment programs) (16). Variability in vectoring capacity and limited understanding of the tick's immune response (particularly with regard to arthropod–microbe interactions for bacteria) have impeded control efforts (17). Although current knowledge is limited, vaccines against ticks are being developed (18). *A. marginale* infections in cattle and buffalo have previously been recorded in different parts of Egypt (19–23). In this study, we report on *A. marginale* in three governorates in the southern part of Egypt, which we targeted because of the lack of research on *A. marginale* and its host species in this part of the country. Specifically, we applied molecular techniques to detect and characterize *A. marginale* infecting cattle, buffaloes, and camels.

## 2. Materials and methods

### 2.1. Study design and research area

**Figure 1:**
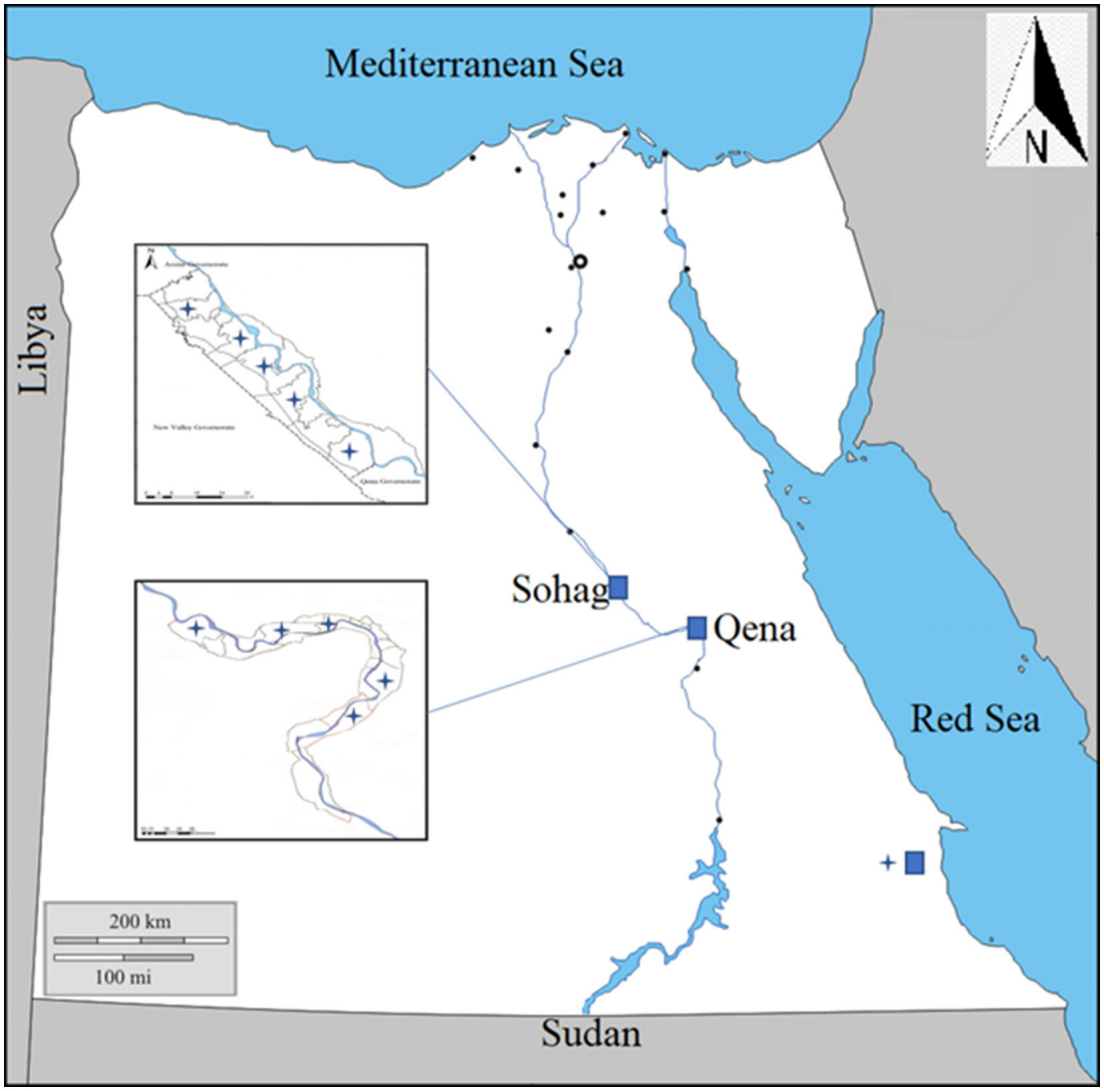
Map showing the southern part of Egypt where blood samples were collected from animals in three governorates, Sohag, Qena, and Red Sea.

This study focused on anaplasmosis infection in local breeds of cattle, buffaloes, and camels of various ages (from 1 to 3 years) and both genders. It was conducted from April 2021 to January 2022, in three southern governorates in Egypt: Sohag, Qena, and Red Sea governates (Figure 1).

### 2.2. Clinical examination

Animals underwent clinical examinations before blood sample collection. The examination involved identifying age and gender and evaluating body mass index, body temperature, heart rate, respiratory rate, and visible mucous membranes. Some cattle were presented with pale mucous membranes, and respiratory disorders were noted in a small number of animals. All animals were infested with ticks, although buffaloes and camels showed no visible specifically associated clinical manifestations.

### 2.3. Collection of samples

Samples were collected from animals selected at random. Small flock breeding mainly determines the species of animal raised by farmers in southern Egypt, which imposes some limitations on sample collection in this region. Accordingly, the number of samples in this study was set so as to provide a clear picture of the epidemiology of the relevant diseases in local animal populations. Whole blood samples were collected from the jugular vein of each animal with clean, sterile vacutainer tubes containing heparin for DNA extraction, as a target for PCR amplification. Samples were kept at −20°C until use.

### 2.4. Detection of control genes and pathogens by PCR

All primers used in this study are listed in Table 1, and the PCR conditions are shown in Table 2. The amplification of bovine β*-actin* for DNA extract was confirmed by amplifying the bovine and camel β*-actin*–encoding genes (housekeeping genes) to ensure that the genomic DNA had been extracted from all samples (24, 25). Negative controls were samples containing nuclease-free water. Electrophoresis of PCR products was performed with 1.5% agarose gel in 1 × Tris–acetate–EDTA (TAE) buffer using a Mupid electrophoresis device (Mupid Co., Ltd., Tokyo, Japan), and bands were visualized through a gel documentation system UV device, WUV-M20 (ATTO Co., Ltd., Tokyo, Japan), after being stained with 5 g/ml ethidium bromide in 1 × TAE.

**Table 1:** The primers for the amplification of target fragments of genes of *Anaplasma marginale*.

**Organism**	**Target gene**	**Primer name**	**Sequence (5^′^ → 3^′^)**	**Expected size (bp)**	**References**
Blood of cattle and buffaloes	*Bovine* β*-actin gene*	FBA	CGCACCACCGGCATCGTGAT	227	(24)
		RBA	TCCAGGGCCACGTAGCAGAG		
Blood of camels	*Camel β-actin gene*	FBC	AGAGCTACGAGCTGCCTGAC	438	(25)
		RBC	GGTTGCCTCAATGTCCATCT		
*Anaplasma marginale*	*groEL*	AMgroES111F1	AGAGCTCGAAGGAAAGAAGTTCATAG	580	(26)
		AMgroEL1557R1	CATGAATACAGCTGCRAGTGACACAG		
		AMgroES67F2	TAATCGCTAAGGAGGCGTAGTC		
		AMgroEL513R2	GTCTTTGGCCCAACTTCCCTTACGCACTG		
*Anaplasma marginale*	*msp5*	AM-49F	GTGTTCCTGGGGTACTCCTATGTGAACAAG	547	(26)
		AM-595R	AAGCATGTGACCGCTGACAAACTTAAACAG		
*Anaplasma marginale*	*msp4*	*msp4*F	GGGAGCTCCTATGAATTACAGAGAATTGTT	854	(27)
		*msp4*R	CCGGATCCTTAGCTGAACAGGAATCTTGC		

**Table 2:** PCR conditions for the amplification of target fragments of genes of *Anaplasma marginale*.

**Target gene**	**PCR condition**
*Bovine β-actin*	$\frac{94^{}\text{C}}{5\text{ min}}\;\to \left[\;\frac{94^{}\text{C}}{30\;s}-\frac{65^{}\text{C}}{30\;s}-\frac{72^{}\text{C}}{30s}\;\right]\;35\times \;\to \frac{72^{}\text{C}}{5\text{ min}}\to 10^{}\text{C }\infty$
*Camel β-actin*	$\frac{94^{}\text{C}}{5\text{ min}}\;\to \left[\;\frac{94^{}\text{C}}{30\;s}-\frac{63^{}\text{C}}{30\;s}-\frac{72^{}\text{C}}{30\;s}\;\right]\;35\times \;\to \frac{72^{}\text{C}}{5\text{ min}}\to 10^{}\text{C }\infty$
*groEL*	$1\text{st round}:\;\frac{95^{}\text{C}}{\;5\text{ min}}\to \left[\;\frac{94^{}\text{C}}{30\;s}\;-\frac{62^{}\text{C}}{30\;s}-\frac{72^{}\text{C}}{1.5\text{ min}}\;\right]\;35\times \;\to \frac{72^{}\text{C}}{5\text{ min}}\to 10^{}\text{C }\infty$
	$2\text{nd round}:\;\frac{95^{}\text{C}}{5\text{ min}}\to \left[\;\frac{94^{}\text{C}}{30\;s}-\frac{65^{}\text{C}}{30\;s}-\frac{72^{}\text{C}}{1\text{ min}}\;\right]\;35\times \to \frac{68^{}\text{C}}{5\text{ min}}\to 10^{}\text{C }\infty$
*msp4*	$\frac{94^{}\text{C}}{5\text{ min}}\to \left[\;\frac{94^{}\text{C}}{30\;s}-\frac{60^{}\text{C}}{30\;s}-\frac{68^{}\text{C}}{1min}\;\right]\;35\times \;\to \frac{68^{}\text{C}}{7\text{ min}}\to 10^{}\text{C }\infty$
msp5^*^	$\frac{95^{}\text{C}}{5\text{ min}}\to \left[\;\frac{95^{}\text{C}}{30\;s}-\frac{{74-68}^{}\text{C}}{30\;s}-\frac{72^{}\text{C}}{1\text{ min}}\;\right]\;36\times \to \frac{72^{}\text{C}}{5\text{ min}}\to 10^{}\text{C }\infty$

^*^Annealing with 0.2°C incremental decreases until reaching the final annealing temperature of 68°C.

### 2.5. PCR amplification and DNA extraction

For this study, 250 samples (from 100 cattle, 75 water buffaloes, and 75 camels) were analyzed by PCR for the presence of *Anaplasmataceae*, specifically *A. marginale*. The animals varied in breed, age, and gender, with most showing no signs of severe disease when samples were collected using commercial extraction kits (Wizard^®^ Genomic DNA Purification Kit, Promega, Madison, WI, USA). DNA was then extracted from whole blood samples. *A. marginale* was detected by screening using nested PCR amplification of the heat-shock protein *groEL* gene using the relevant primers (26). Selected *A. marginale*-positive samples were also subjected to conventional PCR targeting the *msp4* and *msp5* genes (26, 27). The PCR was performed with a total volume of 10 μl, using Tks Gflex DNA Polymerase (TaKaRa), 10 pmol each of the forward and reverse primers, and nuclease-free water. A template (1 μl DNA) was used. The PCR conditions are shown in Table 2. A negative control containing nuclease-free water was added to each PCR. The electrophoresis of the PCR products was performed using 1.5% gel and 1 × TAE buffer. The observation was made using a gel documentation system UV device, WUV-M20 (Atto Co., Ltd.), after the gel was stained with 5 μg/ml ethidium bromide in 1 × TAE.

### 2.6. Sequence and data analysis

The selected *A. marginale groEl, msp4*, and *msp5* genes were subjected to PCR or 50 μl mixtures for sequence analysis. The amplicons were purified using a NucleoSpin Gel and PCR Clean-up kit (Macherey-Nagel, Leicestershire, Duren, Germany), following the manufacturer's protocol. Sequence readings were compared to sequences of reported isolates from a gene bank. A maximum-likelihood phylogenetic tree was constructed using MEGAX software (28), with bootstrap values estimated using 1,000 replicates based on Kimura's two-parameter substitution model (29).

## 3. Results

### 3.1. DNA confirmation and identification

A total of 250 blood samples from cattle, buffaloes, and camels were collected from three governorates in southern Egypt. All 250 samples (100%) were confirmed to contain DNA, as they exhibited bands at the expected 227 bp for cattle and buffalo. The expected 438 bp for camels with the β*-actin* gene demonstrated that DNA had been successfully extracted from all samples.

All samples were then subjected to nested PCR to detect the presence of the *A. marginale groEL* gene. The prevalence of *A. marginale* was 75 out of 250 (30%) samples. By species, 61 out of 100 (61%) cattle were *A. marginale* positive, while 9 out of 75 (12%) buffaloes and only 5 out of 75 (6.66%) camels were *A. marginale* positive (Table 3). All samples positive for the *A. marginale groEL* gene were further examined for two additional genes (*msp4* and *msp5)* to provide an enhanced degree of specificity for the identification of *A. marginale*.

**Table 3:** Detection of *Anaplasma marginale* in cattle, buffaloes, and camels from southern Egypt based on PCR detection in blood samples.

**Species**	**Number of animals**	**Number of negative**	**Number of positive**	**Percent positive**
Cattle	100	39	61	61.00 %
Buffalo	75	66	9	12.00 %
Camel	75	70	5	6.66 %
Total	250	175	75	30.00 %

Furthermore, a higher prevalence of *A. marginale* infection was found in Qena than in Sohag and Red Sea governates. We found no sex difference in any species in this study, based on the relative prevalence of *A. marginale* in males and females. Further investigations of risk factors should encompass univariate and multivariate analyses targeting animal and farm levels. Even so, we found a high prevalence (36% infection rate) in young animals (1 year old or less) relative to the adult animals. The breeding system also appears to be associated with the risk of *A. marginale* infection. Individually, bred animals had a lower infection rate than intensively bred animals (25 vs. 33.3%; Table 4).

**Table 4:** *Anaplasma marginale* infection in cattle, buffaloes, and camels in three governorates in southern Egypt.

**Factors**	**Locations**						**Age**						**Gender**				**Breeding system**			
	**Sohag**		**Qena**		**Red See**		**1 year**		**2 years**		**3 years**		**Male**		**Female**		**Individual**		**Intensive**	
	**N**	**%**	**N**	**%**	**N**	**%**	**N**	**%**	**N**	**%**	**N**	**%**	**N**	**%**	**N**	**%**	**N**	**%**	**N**	**%**
Number of positive-testing animals	30	30	40	40	5	10	18	36	30	30	27	27	15	30	60	30	25	25	50	33.3
Number of negative-testing animals	70	70	60	60	45	90	32	64	70	70	73	73	35	70	140	70	75	75	100	66.7
Total number of tested animals	100		100		50		50		100		100		50		200		100		150	

N = Number, % = Percent.

### 3.2. Sequence analysis

**Figure 2:**
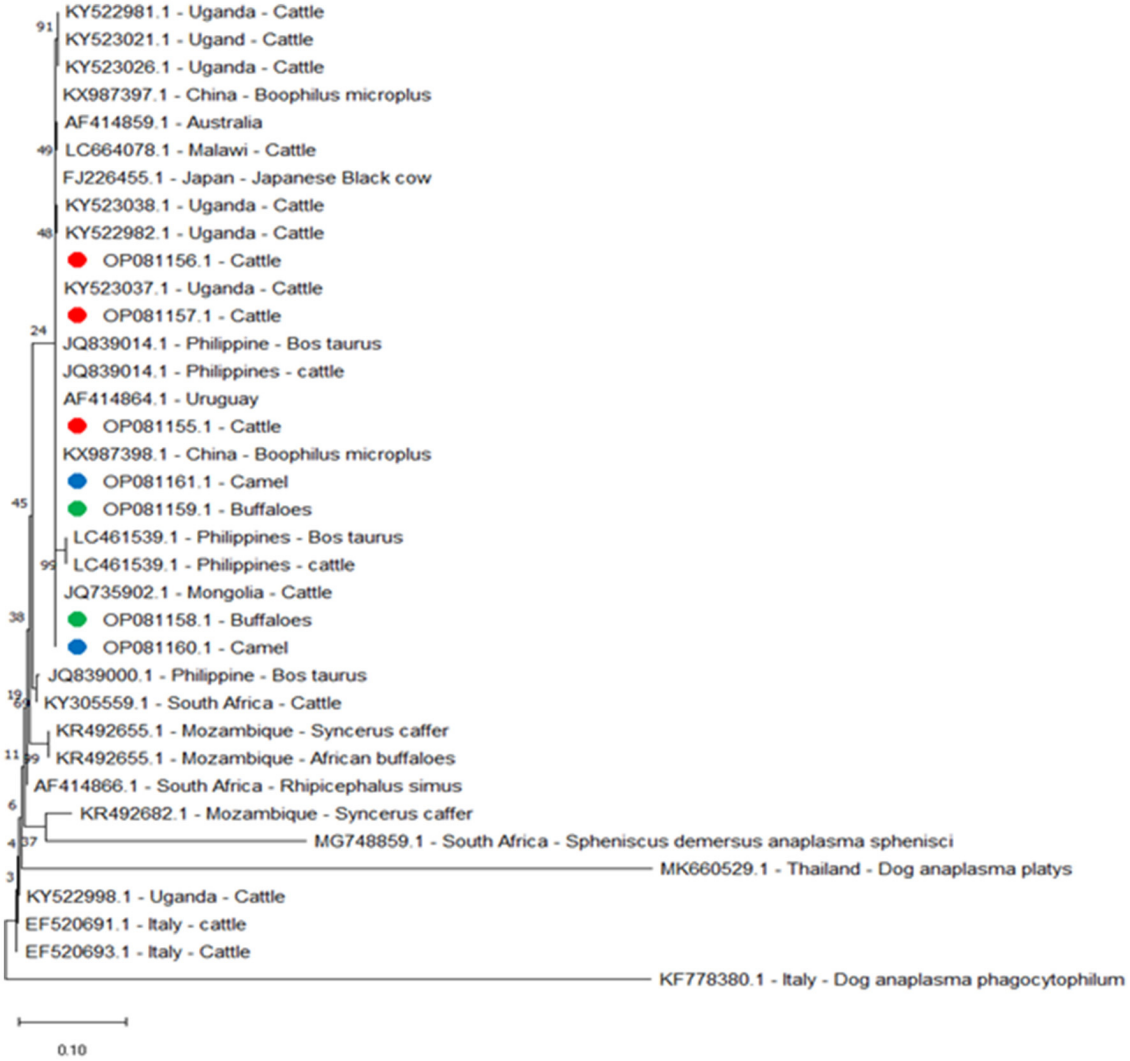
Phylogenetic relationships based on the sequences of the *heat-shock protein* (*groEL*) gene from *A. marginale* using the maximum-likelihood method and Kimura's two-parameter model, with branch lengths measured in the number of substitutions per site. The tree is depicted to scale. The percentage of trees in which the connected taxa clustered together is displayed next to the branches. Red, green, and blue circles represent *A. marginale* obtained in the present study.

**Figure 3:**
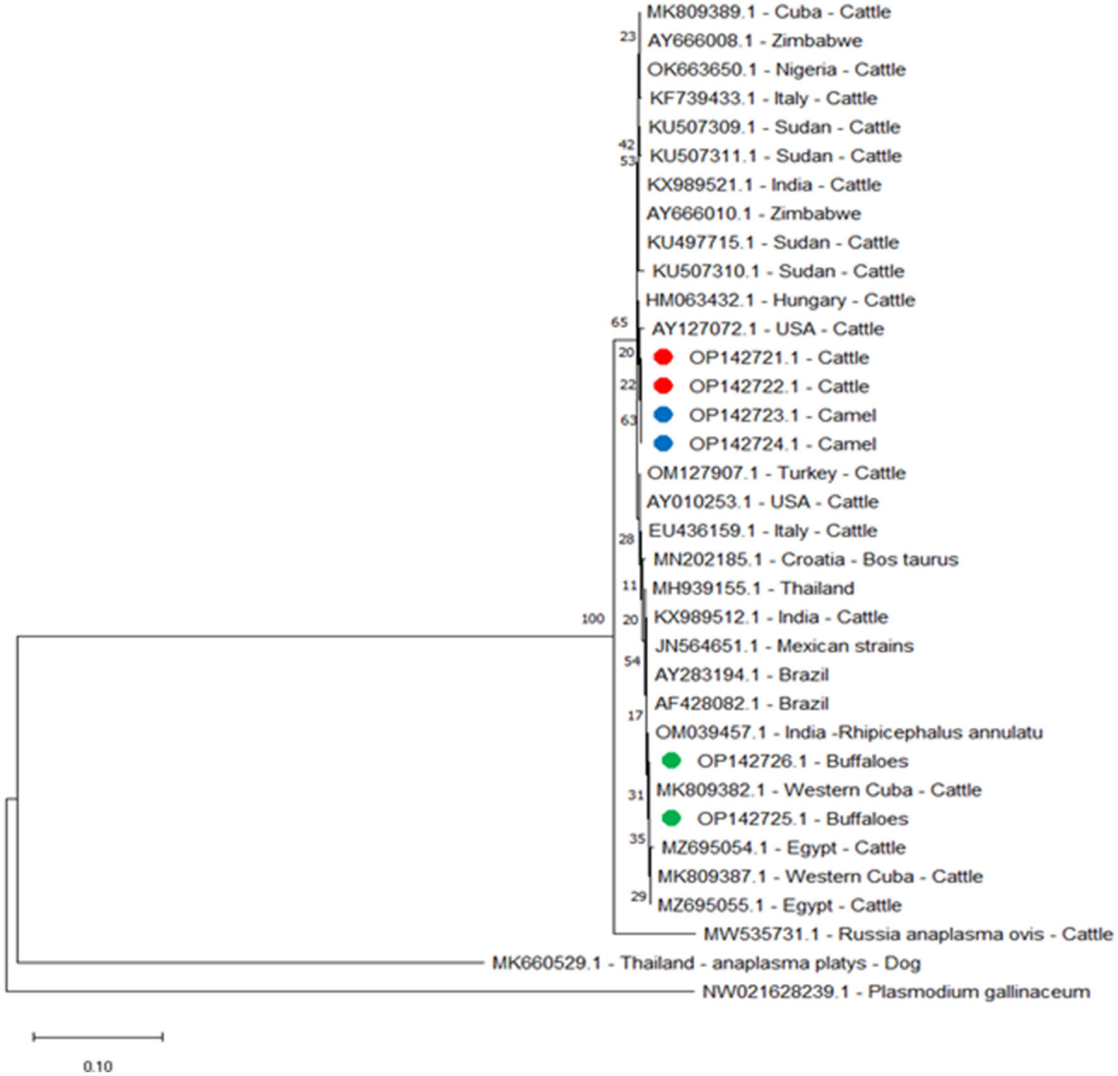
Phylogenetic relations of *A. marginale* using the maximum-likelihood method and Kimura's two-parameter model based on *major surface protein 4* (*msp4*) gene sequences. The percentage of trees in which the connected taxa clustered together is displayed next to the branches. Branch lengths are expressed in terms of the number of substitutions per site, and the tree is drawn to scale. Red, green, and blue circles represent *A. marginale* obtained in this study.

**Figure 4:**
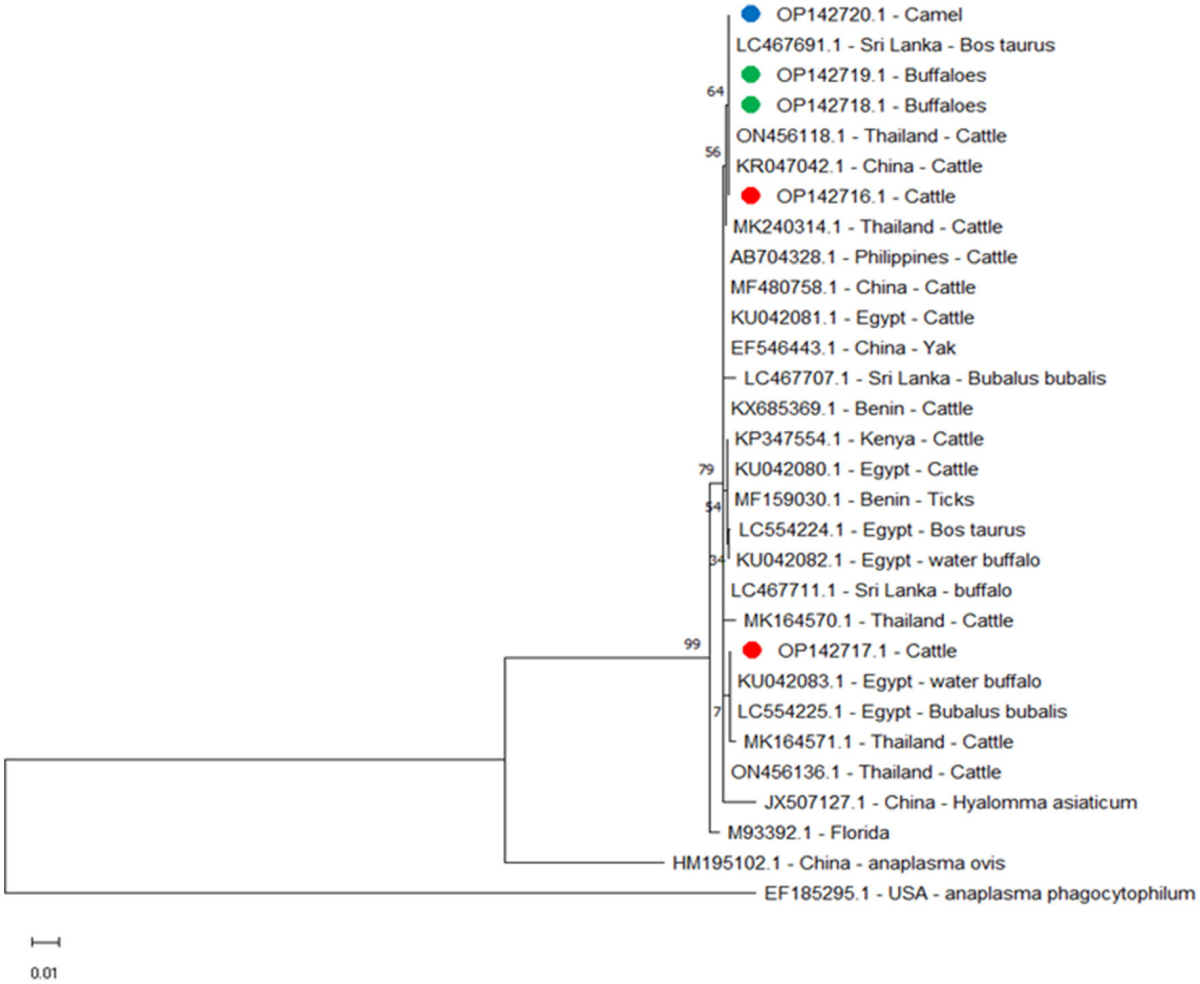
Phylogenetic relationships of *A. marginale* using the maximum-likelihood method and Kimura's two-parameter model based on *major surface protein* 5 (*msp5*) gene sequences. The percentage of trees in which the associated taxa clustered together is shown next to the branches. The tree is drawn to scale, with branch lengths measured in the number of substitutions per site. Red, green, and blue circles represent *A. marginale* obtained in this study.

The *A. marginale* heat-shock protein *groEL* gene and major surface proteins *msp4* and *msp5* genes were sequenced for phylogenetic analysis and genotyping in cattle, buffaloes, and camels from three different governates in southern Egypt. All sequences were also submitted to a gene bank, and the following accession numbers can be used to access them: for the *groEL* gene (cattle: OP081155.1, OP081156.1, and OP081157.1; buffalo: OP081158.1 and OP081159.1; camel: OP081160.1 and OP081161.1); *msp4* gene (cattle: OP142721.1 and OP142722.1; camel: OP142723.1 and OP142724.1; buffalo: OP142725.1 and OP142726.1); and *msp5* gene (cattle: OP142716.1 and OP142717.1; buffalo: OP142718.1 and OP142719.1; camel: OP142720.1). Phylogenetic analysis established the relationships for *A. marginale* with the sequences identified for this study, and various isolates from other countries or other geographic locations in Egypt (Figures 2–4).

A phylogenetic tree was constructed to compare the *groEL* gene for cattle, buffaloes, and camels with amplicons separated from other reported isolates. We found the following alignment identities: 100% to the Philippines, JQ839014.1 and LC461539.1; Malawi, LC664078.1; China, KX987398.1; Uganda, KY523021.1; 99.79% to Japan, FJ226455.1; and 98.07% to Mozambique, KR492655.1 (Figure 2).

For *msp4* amplicons from this study for buffaloes, OP142725.1 and OP142726.1 were identified by the following alignment identities: 100% with amplicons isolated in Western Cuba MK809382.1 and Cuba MK809389.1; 99.88% with Western Cuba MK809387.1; 99.63% with Thailand MH939155.1; 99.38% with USA AY010253.1; 99.25% with Italy EU436159.1; 99.13% with USA AY127072.1; and 99% with Zimbabwe AY666010.1, India KX989521.1, and Hungary HM063432.1. However, for cattle and camel *msp4* amplicons from this study, cattle OP142721.1 and OP142722.1 and camel OP142723.1 and OP142724.1 did not show 100% identity with any amplicon data in the gene bank. They showed 99.75% identity with Zimbabwe AY666010.1 and Hungary HM063432.1; 99.63% identity with India KX989521.1 and Sudan KU497715.1; 99.50% with USA AY010253.1; 99.38% with USA AY127072.1; 99.25% with Italy EU436159.1; 99.13% with Thailand MH939155.1; and 99.00% with Western Cuba MK809382.1 and Cuba MK809389.1. The phylogenetic tree for the *msp4* gene showed that the amplicons from this study for cattle and camel were clustered in a single branch and closely related to a separate branch for other reported isolates from cattle in Sri Lanka and China (Figure 3).

We compared *msp5* amplicons from this study for cattle OP142716.1, buffalo OP142718.1 and OP142719.1, and camel OP142720.1 with amplicons from other reported isolates and found alignment identities of 100% with Sri Lanka LC467691.1 and China KR047042.1; 99.61% with Egypt LC554225.1 and KU042081.1, Philippines AB704328.1, and Benin KX685369.1; 99.59% with Thailand MK240314.1; 99.42% with Egypt LC554224.1 and KU042080.1 and Kenya KP347554.1; 99.40% with Thailand MK164571.1; and 97.86% with the USA M93392.1 (Figure 4).

## 4. Discussion

In this study, we addressed a paucity of complete data on *Anaplasma* species in southern Egypt distributed among cattle, buffaloes, and camel populations. *A. marginale* infection may be more common than previously believed, possibly due to misdiagnosis and undetected carrier animals. We suggest that more detailed information on the distribution of anaplasmosis in southern Egypt is urgently required.

Anaplasmosis is a tickborne rickettsial disease that can adversely affect livestock health and performance worldwide (30). Anaplasmosis reportedly incurs an average cost of $793 per head of cattle, 54% of which can be attributed to death, followed by 15% attributable to treatment, 14% to weight loss, 8% to chronic disease, and 9% to abortion (31). Previous studies in Egypt have shown a wide distribution of *A. marginale* in cattle and water buffalo; however, the data are still incomplete for camels. In Egypt, *A. marginale* is the second-most common tickborne disease in cattle (21.2%); the infection rate in buffaloes is 37.5%, and dromedaries have reportedly been infected with *Babesia* (11.0%), *Theileria* (71.8%), and *Anaplasma* species, as well as *C. burnetti* (20.8%) and *Rickettsia spp*. (31.9%) (32). For anaplasmosis in Egypt, the highest proportions of seropositive animals have been reported in Gharbia (100%), Suez (83.3%), and Port Said (33.3%), whereas the lowest proportions have been recorded in Sohag (4.7%) and Aswan (5.2%) (23). This study was performed to determine the presence of *A. marginale* in cattle, buffaloes, and camels in southern Egypt. The discovery of high prevalence rates of *Piroplasma* and *Anaplasmataceae* among animals that appeared to be in good health—when considered together with the recent rise in international animal trading—suggests the possibility of new genotypes of infections emerging and re-emerging in Egypt following a spread of pathogens from surrounding endemic countries (33, 34). Buffalo from southern Egypt show lower infection rates than cattle from similar areas, and these results may indicate a natural resistance against *A. marginale* in Egyptian buffalo. Previous studies have also demonstrated that water buffalo may show reduced infectivity and cellular replication for this pathogen, resist natural tick infestation, and have a reduced potential for transmitting tickborne diseases (35). The immune system can protect buffalo against high rickettsia levels and related diseases in their acute phase (36). Furthermore, we found that camels had a lower infection rate than cattle and buffaloes, with only five out of 75 camels (6.66%) positive for *A. marginale*. At least four anaplasma species (*A. marginale, A*. *platys, A. phagocytophilum*, and *Candidatus A. camelii*) have been identified as infecting *Camelus dromedarius*. However, infection with *A. marginale* in camel was detected primarily by conventional blood testing with stained blood smears, whereas other *Anaplasma* species in camel were either identified serologically or molecularly (37–40), using *Anaplasmataceae* 16S rRNA-based amplification, sequencing, and phylogenetic tree construction for *A. phagocytophilum, A. platys, A. ovis*, and *Candidatus A. camelii* (41). In only one study, in the Riyadh region of Saudi Arabia, have Arabian camels been shown to have *Anaplasma* species, by amplifying the particular *msp5* gene; the pathogen species was determined to be *A. marginale* (42). To the best of our knowledge, this study presents the first report on using molecular methods and phylogenetic analysis to identify *A. marginale* in dromedary camels in southern Egypt.

Based on these epidemiological results and the genetic variation of *A. marginale* detected in loci different from previous studies (43, 44), we conclude that the prevalence and epidemiological characteristics of *A. marginale* infection are closely related to its geographic location.

Major surface protein genes are under selective pressure from the host immune system and play a significant role in the interaction of *Anaplasma* species with host cells (45–47). All *Anaplasma* species studied thus far have orthologs of the immunodominant outer membrane protein *msp4* (46). Both prokaryotes and eukaryotes have a highly conserved housekeeping gene called g*roEL*. *Ehrlichia, Rickettsia*, and *Anaplasma* species are all members of the *Rickettsiales* bacterial family, and this gene has recently been used in phylogenetic analyses of these species (47, 48). Six membrane surface proteins of the initial bodies of this organism (carriers of epitopes B and T) have been characterized. Major surface proteins have been named and identified as 1a, 1b, 2, 3, 4, and 5 (49); these proteins were recognized by neutralizing antibodies, and they have a strong intermolecular relationship in the membranes of the initial bodies, performing essential functions (36). Genes encoding these proteins have been studied, showing their protein products to have a variable polymorphism. They can be represented in the genome by a single copy gene or by forming part of multigenic families (50). All *Anaplasma* species and all examined isolates of *A. marginale* have shown a single copy of the *msp5* gene present in their genomes. This gene is highly conserved, and it is a strong candidate for diagnosing bovine anaplasmosis, since the *msp5* protein it produces has low structural complexity, is similarly conserved, and elicits high antibody titers (51).

Whenever infected cattle are moved, a new genotype of *A. marginale* is imported to their new location, and this genotype may then spread to susceptible cattle either mechanically or biologically. Few genotypic variations are detected in *A. marginale* isolates from places like Australia where cattle movements are rare (52). Dogs were shown to be carriers of ticks that disseminated *A. marginale* infection to cattle herds, and it is thus likely that physical contact between animals could result in tick transmission from one host to another, spreading tickborne diseases between them (53). The close contact between cattle and buffalo, particularly in the individual breeding system, could be a factor in the transmission of *A. marginale* between the different animal species. On the other hand, there is minimal interaction between camels and other animals; however, camels can be transported by the same vehicles as used to transport cattle and buffaloes, and such vehicles may become a path of infection and contribute to the spread of *A. marginale* in animal populations in southern Egypt and elsewhere.

The prevalence of *A. marginale* is known to vary according to environmental conditions, sample site, vector species, host breed, and breeding system (54). According to our research, intensive breeding systems had a higher infection rate than individual breeding systems (33.3 vs. 25%). This may be because there is more animal contact in intensive breeding systems than in individual breeding systems, making it easier for ticks to spread from one animal to another. Management practices differ from farm to farm in the southern area, where most farms house small numbers of co-reared animals because most farmers implement a multidisciplinary system encompassing pastoral and arable farming. Accordingly, predicting direct effects on disease epidemiology is challenging due to limitations in potential study populations.

Phylogenetic analyses using the *msp4* gene have been used to elucidate the biogeography and evolution of the *Anaplasma* spe*c*ies (46). Phylogenetic analysis based on *msp4* for *A. marginale* showed that the amplicons from this study for cattle and camel cluster in a single branch and have a close relationship with separate branches of other reported animal amplicons. According to one report in 2022, *A. marginale* was detected in camels in southern Egypt using the *msp5* gene; however, that report did not provide any data on phylogenetic analyses of *A. marginale* in camels (55).

The phylogenetic analyses based on *groEL* and *msp5* genes produced very similar results to those on isolates from other locations, possibly due to the unregulated movement of live animals between locations in Egypt for slaughter and marketing. Such local circulation of pathogens should be considered even though the issue of globally circulating tick diseases has gained attention recently with the importation of live animals from other countries to Egypt.

## 5. Conclusion

The findings of this study indicate that *A. marginale* is highly prevalent in *camels*, cattle, and buffaloes in southern Egypt. The identity of the *A. marginale* was confirmed by amplifying the specific *msp4* and *msp5* genes in phylogenetic analysis, which provided new data for *A. marginale* in southern Egypt. According to obtained results, *A. marginale* infection is endemic in different animal species in southern Egypt. It is the first report using three genes for *A. marginale* in *Camelus dromedarius* in southern Egypt.

## Data availability statement

The datasets presented in this study can be found in online repositories. The names of the repository or repositories and accession number(s) can be found in the article or supplementary material.

## Ethics statement

The animal study was reviewed and approved by the Faculty of Veterinary Medicine, South Valley University (10/09.02.2021, 53/13.09.2022).

## Author contributions

HM and TT: conceptualization, design, experiments, formal analysis, investigation, writing of the original draft, project administration, and funding acquisition. HM, AA, and TT: resources, shared materials, and writing – review and editing. All authors contributed to the article and approved the submitted version.
